# In-Hospital Death of Patients with Known Life-Threatening Disease: A Retrospective Analysis

**DOI:** 10.1089/pmr.2024.0051

**Published:** 2024-11-27

**Authors:** A. E. Lijst, D. M. Korst, E. M. Witteman, R. T. M. van Dongen, C. M. P. W. Mandigers

**Affiliations:** ^1^ Department of Palliative Care Consultation Team, Canisius-Wilhelmina Hospital, Nijmegen, The Netherlands.; ^2^ Maastricht University, Maastricht, The Netherlands.; ^3^ Department of Gastrointestinal and Liver Diseases, Canisius-Wilhelmina Hospital, Nijmegen, The Netherlands.; ^4^ Pain Clinic, Nijmegen, The Netherlands.; ^5^ Department of Internal Medicine, Nijmegen, The Netherlands.

**Keywords:** advance care planning, end of life, overtreatment, palliative care

## Abstract

**Background:**

In the Netherlands, overtreatment at the end of life and the high incidence of in-hospital death have led to discussions on how to improve advance care planning.

**Objectives:**

To investigate in-hospital deaths at the Canisius-Wilhelmina Hospital, Nijmegen, the Netherlands in 2019 and 2022 of patients previously diagnosed with at least one life-threatening disease, who received symptom-oriented treatment within seven days of admission.

**Design:**

Retrospective study.

**Measurements:**

Characteristics of the patient population and their final hospital admission were analyzed.

**Results:**

In the Canisius-Wilhelmina Hospital, 216 and 180 patients died in 2019 and 2022, respectively, who were treated for at least one life-threatening disease and who received symptom-oriented treatment within seven days of admission. Most of these patients were referred to the emergency room from home. They were admitted for median three days before their in-hospital death. Advance care was documented in 1% and 2% of cases in 2019 and 2022, respectively.

**Conclusion:**

A significant number of in-hospital deaths at the Canisius-Wilhelmina Hospital in 2019 and 2022 could be considered expected deaths. Furthermore, advance care planning was rarely documented in these cases. Whether improvement of advance care planning could reduce the number of deaths occurring in-hospital should be the subject of further investigation.

## Key Message

In patients with a life-threatening disease, in-hospital death is high, yet advance care planning is rarely documented. We advise to incorporate advance care planning earlier in patient treatment in order to improve patient care in the last phase of life.

## Introduction 

In an era of increasing treatment options for an aging population, it is crucial to recognize the final stage of a patient’s life and to discuss that patient’s wishes. When this is not done, overtreatment and even inadvertent in-hospital death are likely to occur.^1–3^ In the final phase of life, it is important to determine what is still meaningful in the patient’s life and to choose appropriate care, incorporating shared decision making with patient and caregiver.^4^ Appropriate care also contributes to sustainable care, as advised by the Scientific Council for Government Policy in the Netherlands 2021.^5^

The quality of end-of-life care in the Netherlands has improved in 2021 compared with 2017, as can be derived from the demonstrated decrease of the use of acute care during the last months of life and an increase in the number of patients dying at home.^6,7^

Palliative care aims at improving the quality of life for patients and their families, facing a life-threatening condition. Here, advance care planning is indispensable, as life goals and appropriate care must be continuously reassessed on an individual basis.^8^ Any health care professional can provide palliative care, with or without consulting a palliative care team.

The palliative care consultation team at the Canisius-Wilhelmina Hospital (CWZ) in Nijmegen, the Netherlands, retrospectively investigated in-hospital death of patients with pre-existing severe illness and whether there was prior advance care planning. We analyzed all deaths occurring in 2019 and 2022 in patients who received symptom-oriented treatment within seven days of admission and were known to have at least one disease from which death was expected within one year. In addition, we discuss how to improve palliative care and reduce the number of expected deaths occurring in-hospital.

## Material and Methods

### Study design

Since 2009, medical records of all deceased patients at the CWZ, Nijmegen, the Netherlands, are studied by the “Necrology Committee” consisting of a group of external medical specialists. They categorize deceased patients based on the time since admission when treatment was no longer curative but symptomatic, intended to provide relief in the final stage of life.^9^ We retrospectively selected patients who received this symptom-oriented treatment either on admission or within the first seven days of hospitalization.

We analyzed patient cohorts from 2019 and 2022 because the intervening years were significantly affected by the COVID-19 pandemic, which had a major impact on our hospital mortality rates.

The patient records were studied by four investigators, namely a medical student, a nurse practitioner, an internist-hematologist-medical oncologist, and an anesthesiologist-pain specialist, all from the palliative care consultation team at the CWZ. They used the surprise question to define patients with an expected death: “Would I be surprised if this patient were to die within 12 months?”.^10^

The researchers included only patients who were treated by the medical specialist(s) in the CWZ for at least one disease, from which death within a year would not surprise any of the four investigators (hereafter referred to as ‘life-threatening disease’).

### Measurements

Demographic and medical data including life-threatening diseases were recorded for all included patients. Data were collected on medical care received prior to the last hospital admission, whether treatment limitations (e.g., do not resuscitate, do not intubate) were documented in the CWZ, and the number of admissions to the CWZ in the last year before death. Investigators examined where the patients came from, when and how they were referred and for what complaint. They determined whether the last hospital admission was due to the pre-existing life-threatening disease and the duration of admission. Finally, they searched for any notes on advance care planning preceding the last hospital admission and previous involvement of the palliative care consultation team.

All four investigators answered the question whether this last hospital admission could have been prevented. A potentially preventable admission was defined as an admission when death was expected due to the known life-threatening disease, for example, an admission due to pneumonia in a patient in the terminal stage of an extensively metastasized pancreatic carcinoma. In contrast, a last hospital admission was defined as not potentially preventable if there was an unexpected acute reason for admission, whether or not related to the pre-existing life-threatening disease, for example, an admission due to a femoral fracture in a patient with end-stage of Parkinson’s disease. When either the patient or relatives had a strong wish for treatment in hospital or even dying in hospital, this last hospitalization was defined as not potentially preventable. Consensus on these two categories was reached through joint discussion.

## Results

**FIG. 1.**
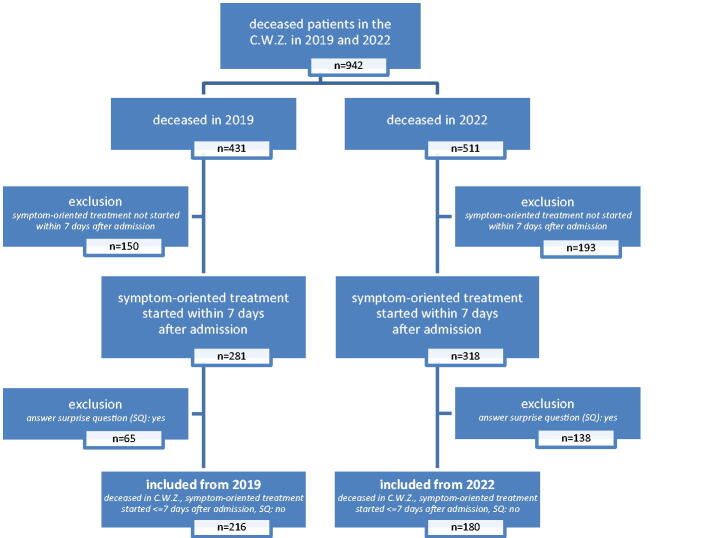
Study flowchart. SQ, ‘Would I be surprised if this patient were to die within 12 months?’.^10^

In the CWZ, 216 and 180 patients died in 2019 and 2022, respectively, who were known to have at least one disease from which death within a year would not surprise anyone and who were offered a symptom-oriented treatment within seven days of admission. Figure 1 shows the flowchart of the study.

In Table 1, an overview of the characteristics of the patient population and their last hospital admission is presented.

**Table 1.** Characteristics of the Patient Population and Their Last Hospital Admission

	2019		2022	
	Number	Percentage	Number	Percentage
Included patients (study population by year)	216	100%	180	100%
Sex				
Male	114	53%	94	52%
Female	102	47%	86	48%
Median age in years (min^a^–max^b^)	79,0 (24–100)		77,5 (32–100)	
Median number of life-threatening diseases (min^a^–max^b^)	1 (1–4)		1 (1–4)	
Most life-threatening disease				
Hemato-/oncological	65	30%	57	32%
Pulmonary	55	25%	50	28%
Cardiac	46	21%	39	22%
Neurological	30	14%	20	11%
Other	20	10%	14	7%
Professional care before admission				
Present	94	44%	107	59%
Treatment limitations before admission				
Present	123	57%	125	69%
Median number of admissions in the year preceding the last admission (min^a^–max^b^)	0 (0–8)		1 (1–8)	
Patients came to the hospital from				
Home	183	85%	154	86%
Nursing home	33	15%	26	14%
Patients came to the hospital by				
Referral by a doctor	165	76%	135	75%
Self-referral	51	24%	45	25%
Arrival at the emergency department				
During a working day	95	44%	95	53%
In the evening, night, or weekend	121	56%	85	47%
Most frequent symptom to referral				
Dyspnea	118	49%	95	49%
Reason to be admitted				
Known life-threatening disease	124	57%	98	54%
Median duration of admission in days (min^a^–max^b^)	3 (0–19)		3 (0–16)	
Death within 48 hours after arrival at emergency department	60	28%	44	24%
Advance care planning before last admission				
Present	3	1%	3	2%
Consultation of palliative care team				
Present	72	33%	62	34%
Median duration between consultation of palliative care team and death in days	1		1	
Potentially preventable last hospital admission				
Yes	152	70%	117	65%

^a^ min, minimum.

^b^ max, maximum.

Most individuals were over 77 years of age and had a maximum of four life-threatening diseases. Compared with the 2019 cohort, patients in the 2022 cohort had more often professional care from a nurse or caregiver before the last hospital admission and had more frequently a treatment limitation documented in CWZ. Patients were admitted median 0 and 1 times in the year preceding the last hospital admission, with a maximum of eight times in both years.

The majority of patients presented to the emergency department (ED) from their own home, most often after referral by a doctor. About half of them presented on a weekday during daylight hours, with dyspnea as the most common reason for referral. Over half of the patients were admitted because of their previously diagnosed life-threatening disease. The median duration of this last hospital admission was three days in both cohorts, with a quarter of them dying within 48 hours after an ED visit.

Advance care planning before the last hospital admission was documented in the CWZ for only three patients in both 2019 and 2022.

The palliative care consultation team was consulted in 2019 and 2022 in one-third of the patients, with a median of one day before the patient’s death in both years. We assessed in-hospital death to be potentially preventable in 70% and 65%, respectively.

## Discussion

This retrospective analysis of potentially preventable in-hospital death in the CWZ in the Netherlands in the years 2019 and 2022 demonstrated that advance care planning was largely lacking.

Most of these patients were elderly individuals known to have serious, life-threatening hemato-/oncological, pulmonary, cardiac, and/or neurological diseases. Before admission many already received professional care and had treatment limitations documented in their hospital files, more often in the last year examined. More than half of the patients were referred and admitted because of their previously diagnosed life-threatening disease, with dyspnea as the most frequent symptom. The median length of hospitalization prior to death was only three days.

What struck us most in our study is the enormous lack of advance care planning despite the facts that our patients were known to have life-threatening disease(s) and treatment limitations had frequently been documented. Advance care will greatly improve the quality of the last phase of a patient’s life as it allows medical practitioners to choose appropriate care in shared decision with the patient and family. Advance care planning should be initiated on time and should be repeated if the patient’s condition changes and documented in the patient file.^8^

The initiative for discussion of life goals and appropriate care can be taken by health care professionals as well as the patient or family members. A patient may wait for others to take this initiative for a variety of reasons, such as anxiety surrounding death or not wanting to be too demanding for health care providers. Similarly, loved ones may also wait because they think, often incorrectly, that talking about how to live life before an impending death is burdensome for the patient. Furthermore, health care providers from primary and hospital care settings can be reluctant to approach the topic, either because they do not feel competent or because they believe that advance care planning is the responsibility of another doctor, especially in cases of insufficient or unsatisfactory communication. Previous research by our team found that both professional care and treatment limitations before hospital admission are predictive of imminent death and could therefore lead to advance care planning.^11^

In our study, patients were admitted a median of 0 and 1 times in the last year before their last hospital admission in 2019 and 2022, respectively, with a maximum of up to 8 times. If a patient is frequently admitted for a known life-threatening illness, we think that the initiative for advance care planning lies with the medical specialist in collaboration with the general practitioner. Both should recognize a patient’s last phase of life and act accordingly by initiating advance care planning.

In our hospital, the multidisciplinary palliative care team can be consulted for patients in whom the answer to the surprise question is ‘no’ and for whom specialized care focusing on the physical, psychological, social and/or spiritual dimensions may be needed. In both the 2019 and 2022 cohort, the palliative care team was consulted for one-third of the patients, with a median time between consultation and death of only one day. Care was sought to provide comfort in the dying phase, long after the moment where advance care planning might have prevented the last hospital admission. It is reported that hospital-based palliative care teams are often more crisis-oriented, rather than prevention-oriented.^12^

In a cross-sectional study regarding 8.789 patients admitted to 48 Dutch hospitals in the year 2021, it was estimated that one-third of these patients had a life expectancy of less than one year, suggesting that they would be candidates for palliative care. However, only 2% of them were actually seen by a palliative care team.^13^

These observations suggest that advance care planning is not standard of care yet, although it is known to be beneficial for patients, their family, and the health care system. Discussions regarding optimal implementation are currently ongoing.^14,15^ Our study does not show improvement in advance care despite lessons that could have been learned from the COVID-19 pandemic.^16^

In addition to the discussed lack of advance care, there are other possible causes of hospitalization in the final phase of life, contributing to in-hospital death of patients known to have a life-threatening disease. From a practical point of view, there may be insufficient possibilities for adequate end-of-life care in the primary care setting. The primary physical symptom in our study, dyspnea, has various causes and may be associated by anxiety and severe suffering, both of which can lead to hospital referral.^17,18^ Other factors are lack of time to arrange additional nursing care on short notice, pressure of the family on the general practitioners to refer the patient due to unrealistic expectations, and inadequate information among health care providers due to poor communication.

Due to the retrospective character of our study, we encountered several limitations and drawbacks. First of all, we could not compare our patients with a similar series of patients who died at home or in a hospice because we did not have access to the general practitioners’ records of this patient group.

In addition, although treatment limitations were documented in most patients’ files, we could not find notes of advance care planning as discussed with the treating hospital physician.

Furthermore, we used the surprise question for selecting patients with life-threatening disease: “Would I be surprised if this patient were to die within 12 months?”.^10^ If the answer is “no,” this identifies patients whose condition may rapidly deteriorate.^8^ The low predictive value of this question can be improved by adding a second surprise question: “Would I be surprised if this patient were still alive in 12 months?”, also known as the double surprise question.^19^ For practical reasons, we used the surprise question instead of other validated prognostic or supportive care indicators tools in our diverse patient group. This may have led to selection bias.

Finally, an unexpected, sudden increase in physical symptoms (e.g., dyspnea) may lead patients or their caregivers to urge referral, which may be reinforced by the absence of an adequate treatment plan at home and despite a limited chance for reversibility. These factors were not available in our hospital records.

In conclusion, we brought to light the severe lack of advance care planning in severely ill patients in the CWZ in both 2019 and 2022, presumably contributing to a large number of patients being admitted in their final days of life. We expect that implementation of advance care planning after recognizing the final phase of a patient’s life can lead to a better quality of last life. It provides relief for both the patient and family and may contribute to sustainable care in the future. We advise to incorporate advance care planning earlier in patient treatment in order to improve care in the last phase of life.

## Abbreviations Used

COVID-19: Coronavirus Disease 2019
CWZ: Canisius-Wilhelmina Hospital
ED: emergency department
SQ: surprise question
